# A new model of self-resolving leptospirosis in mice infected with a strain of *Leptospira interrogans* serovar Autumnalis harboring LPS signaling only through TLR4

**DOI:** 10.1038/emi.2017.16

**Published:** 2017-05-24

**Authors:** Bili Xia, Le Sun, Xia Fan, Haihan Xiao, Yongzhang Zhu, Jinhong Qin, Chengsong Cai, Wei Zhao, Yung-Fu Chang, Yan Zhang, Xiaokui Guo, Ping He

**Affiliations:** 1 Department of Microbiology and Immunology, Institutes of Medical Science, Shanghai Jiao Tong University School of Medicine, Shanghai 200025, China; 2 Shanghai Public Health Clinical Center, Fudan University, Shanghai 201508, China; 3 Department of Laboratory Medicine, Affiliated Hospital of Hangzhou Normal University, Hangzhou 311121, Zhejiang Province, China; 4 Department of Population Medicine and Diagnostic Sciences, College of Veterinary Medicine, Cornell University, Ithaca, NY 14853, USA

**Keywords:** leptospires, lipopolysaccharide, mouse model, TLR4

## Abstract

Leptospirosis is an emerging worldwide zoonosis caused by pathogenic *Leptospira* spp. Our understanding of leptospirosis pathogenesis and host immune response remains limited, while mechanistic studies are hindered by a lack of proper animal models and immunological reagents. Here we established a murine model of acute and self-resolving leptospirosis by infecting 10-week-old C57BL/6 mice with *Leptospira interrogans* serovar Autumnalis strain 56606v, with characteristic manifestations including jaundice as well as subcutaneous and pulmonary bleeding, but no kidney lesions. We also verified that the lipopolysaccharide (LPS) of strain 56606v signaled through a TLR4-dependent pathway in murine bone marrow-derived macrophages (BMDMs), rather than the previously reported TLR2. In addition, upon infection with *Leptospira* strain 56606v, TLR4^−/−^ C57BL/6 mice presented more severe jaundice and liver injury as well as higher bacterial loads than WT mice but milder pulmonary hemorrhaging. Molecular studies showed that leptospirosis-related bleeding coincides with the temporal kinetics of iNOS production, while jaundice and liver injury are probably due to insufficiently controlled bacterial loads in the liver. These results suggested that TLR4 is essential in mediating host leptospiral clearance and, to some extent, is associated with pulmonary and subcutaneous hemorrhage, probably through downstream inflammatory mediators, iNOS in particular. Overall, our murine model using immunocompetent mice might facilitate future studies into the pathogenesis of jaundice and bleeding in leptospirosis. Meanwhile, our study suggests the prospect of combining antibiotics and immunosuppressants in the treatment of severe leptospirosis presenting with pulmonary hemorrhage.

## INTRODUCTION

Leptospirosis has been recognized as an emerging worldwide zoonosis that is largely endemic in tropical areas with frequent flooding and especially in areas with poor sanitation.^1, 2^ Leptospirosis is caused by pathogenic *Leptospira* spp., which naturally reside in maintenance hosts including rodents, and can be transmitted to accidental hosts including humans through direct or indirect skin and mucosa contact with the urine of infected animals.^3^ The severity of human disease caused by leptospiral infection ranges widely from asymptomatic or subclinical to the characteristic features of severe leptospirosis, which include myalgia, jaundice, pulmonary hemorrhage and renal injury.^2, 3^

Animal models constitute an essential tool in studying the pathogenesis of leptospirosis. Multiple animal models have been employed in studying the pathogenesis of leptospiral infection, with varied disease forms among different hosts mostly depending on host susceptibility to *Leptospira* and the virulence traits of the bacterial strain.^4, 5, 6^ Guinea pigs and hamsters have been successfully used as animal hosts because their leptospirosis disease course bears a close resemblance to that in humans.^6, 7, 8^ Most studies are conducted using golden Syrian hamsters as models of infection, reproducing human leptospirosis with manifestations of jaundice, hemorrhage and interstitial nephritis.^9, 10^ However, due to a lack of immunological and biological reagents, the hamster model poses obstacles to our understanding of host immune responses to leptospiral infection. In addition, even though mouse models possess the advantage of immunological reagents, the application of a mouse model is hindered by their natural resistance to the disease.^11^ Mouse models for leptospiral infection are mostly confined to mice younger than 6 weeks of age or immunocompromised mice with certain immune response-related genes deleted.^12, 13, 14^ Usually, mice serve as chronic carriers of leptospiral infection, with predominant renal colonization of leptospires leading to interstitial nephritis or kidney failure, but remain systemically asymptomatic or have mild clinical symptoms.^15, 16, 17, 18^

In this study, we established a mouse model of acute leptospiral infection by infecting C57BL/6 mice with *L. interrogans* serovar Autumnalis strain 56606v (Lin4), one of the epidemic *Leptospira* strains in China, isolated from a patient infected with *L. interrogans* in Zhejiang Province, China.^19^ The manifestations of our model included prominent jaundice and hemorrhage into pulmonary and retroperitoneal tissues, which are hallmarks of leptospirosis in humans. Kidney colonization, seen in most mouse reservoirs of chronic infection, is absent in this study, however. The typical findings observed in our murine model suggested that it could be an applicable animal model of leptospiral infection and host immune response recapitulating severe acute human leptospirosis.

Toll-like receptors (TLRs) play a critical role in bacterial recognition and mediating host recognition and immune defense against the leptospires and their outer membrane components including lipopolysaccharide (LPS).^20, 21^ Leptospiral LPS has been shown to be distinctly different from that of enterobacterial species such as *Escherichia coli*, both structurally and functionally.^20, 22^ Nahori *et al.* previously showed in an *in vitro* study that TLR2 was involved in LPS signaling in human cells, while stimulation of murine cytokine production by leptospiral LPS was both TLR2 and TLR4 dependent.^20^ In this study, properties of LPS derived from *L. interrogans* strain 56606v were explored. Meanwhile, TLR4 knockout mice were used to explore the disease pathogenesis.

## MATERIALS AND METHODS

### Ethics statement

The conducts and procedures involved in the animal experiments were approved by the Animal Ethics Committee of Shanghai Jiao Tong University School of Medicine (project number 2016023) and according to Regulations for the Administration of Affairs Concerning Experimental Animals (approved by the State Council of the People’s Republic of China) and Guide for the Care and Use of Laboratory Animals (Department of Laboratory Science, Shanghai Jiao Tong University School of Medicine, laboratory animal usage license number SYXK2013-0050 certificated by Shanghai Committee of Science and Technology).

### Reagents

Chemicals were purchased from Sigma-Aldrich Co. (St Louis, MO, USA) unless otherwise noted. Endotoxin-free phosphate-buffered saline (PBS; pH 7.4), Dulbecco’s modified Eagle’s medium and heat-inactivated fetal bovine serum were purchased from GIBCO (Thermo Scientific, Waltham, MA, USA). *Leptospira* Ellinghausen-McCullough-Johnson-Harris (EMJH) was purchased from BD Difco (Baltimore, MD, USA). The ultrapure LPS from *E. coli* O55:B5 (*E. coli* LPS) was ordered from Sigma-Aldrich. The synthetic triacylated lipoprotein Pam3CSK4 was purchased from InvivoGen (San Diego, CA, USA). TRIzol LS Reagent, RNase A and DNase I were purchased from Life Technologies (Thermo Scientific).

### Animals

C57BL/6 wild-type (WT) mice (female, 10-week-old) were purchased from Shanghai Laboratory Animal Company (SLAC) China. TLR4 knockout (TLR4^−/−^) mice were generated by Dr S. Akira and were backcrossed for eight or more generations to the C57BL/6 background.^23^ Mice deficient in TLR2 (TLR2^−/−^) on a C57BL/6 background (Stock NO N000157) were purchased from Nanjing Biomedical Research Institute of Nanjing University. The mice were housed under specific-pathogen-free conditions and fed with autoclaved food and water in the Department of Laboratory Animal Science, Shanghai Jiao Tong University School of Medicine.

### *Leptospira* infection

The strain used in this study was *L. interrogans* serovar Autumnalis strain 56606v, which was originally isolated from a leptospirosis patient in Zhejiang Province, China.^19, 24^ The challenge inoculum was a virulent, low-passage isolate (passage 2) that can cause acute leptospirosis in golden hamsters and guinea pigs with typical presentations of jaundice and pulmonary hemorrhage. The virulence of the strain was maintained by iterative passage in young golden hamsters and growth in liquid EMJH medium under aerobic conditions at 28 °C to mid-log phase. Just before infection, the bacteria were centrifuged, washed twice in endotoxin-free PBS and counted using a Petroff–Hausser chamber (Electron Microscopy Sciences, Hatfield, PA, USA), then resuspended in endotoxin-free PBS to 10^9^ bacteria per mL. The mice were infected with 2 × 10^8^ leptospires in 200 μL of PBS via the intraperitoneal (ip) route. Negative control animals were intraperitoneally injected with 200 μL PBS. The animals were bled and killed at 8 h, 1, 2, 3, 5, 7, 14 and 28 days postinfection (dpi), respectively. Blood samples were collected through the submandibular venous plexus. Approximately 350 μL whole blood was put into tubes containing 60 μL anticoagulant acid citrate dextrose (ACD), then stored at −80 °C for DNA or RNA extraction, and the rest of the whole blood was processed for serum biochemical analysis. The liver, lungs, spleen, and kidneys of infected and negative control mice were collected and either frozen or ground in liquid nitrogen for RNA extraction or fixed in neutral buffered 4% formaldehyde overnight, processed and stained for histological and immunohistochemical analysis.

### Leptospiral load in blood

Genomic DNA was extracted from *L. interrogans* strain 56606v from both 50 μL animal blood samples and *in vitro* cultures using a DNeasy Blood and Tissue Kit according to the manufacturer’s instructions (Qiagen, Hilden, Germany). The concentration of leptospires in the animal blood was quantified with an ABI 7500 Fast Real-Time PCR System (Applied Biosystems, Foster City, CA, USA) and Power SYBR Green PCR Master Mix (Applied Biosystems). The concentration of the final PCR product of 116 bp (16S rRNA: 5′-AGC ACG TGT GTT GCC CTA GAC ATA-3′ and 5′-GTT GCC ATC ATT CAG TTG GGC ACT-3′)^25^ was determined using a standard curve generated with genomic DNA extracted from 10-fold dilutions of known quantities of *L. interrogans* strain 56606v. The results are presented as the number of leptospires in 1 μL blood. Real-time PCR was performed in duplicate for each DNA extraction. Three mice were used for each time point in each group.

### Pathological and immunohistochemical studies

Tissues (liver, lungs, spleen and kidneys) were fixed in neutral buffered 4% formaldehyde, followed by embedment of the tissues in paraffin and sectioning into 5 μm-thick slices on glass slides. The samples were then dewaxed in xylene and rehydrated, followed by staining according to the hematoxylin and eosin (HE) staining protocol. The evaluation of tissue injury was performed by two experienced board-certified veterinary pathologists blinded to the experimental grouping, who scored the extent of tissue injury into four categories including absent, mild, moderate, or severe injury (score 0–3) as previously described.^26^ For tissue samples from each mouse, fields from each section were randomly selected under × 10 magnification microscopy. The score of tissue injury was recorded (for example, hemorrhage and inflammatory infiltration for characterization of lung injury), and the mean of scores from each section was calculated, while the total injury score was obtained by adding up the scores of each of the categories as previously described.^26^ *L. interrogans* strain 56606v-specific rabbit antiserum was prepared in our lab with modifications to a previously reported procedure.^27^ Immunohistochemical staining of the tissue sections was performed using the EnVision system (Dako, Glostrup, Denmark),^28^ including a serial procedure of dewaxing and rehydration of paraffin-embedded tissue sections, treatment with 3% H_2_O_2_ for 10 min, and incubation of the sections in 0.25% trypsin at 37 °C for 30 min, followed by a second incubation process in *L. interrogans* strain 56606v-specific primary rabbit antibody (1:4000 dilution) for 12 h at 4 °C. After 30 min of EnVision, the tissue sections were then visualized with 3,3′-diaminobenzidine (DAB) and counterstained with modified hematoxylin. For the elimination of bias, the specimen preparations were viewed by a pathologist blinded to the infection status of the animals.

### LPS extraction and purification

*L. interrogans* strain 56606v LPS (L06vLPS) was extracted by the hot phenol-water method as described previously with modifications.^21^ In brief, *L. interrogans* strain 56606v was collected at a density of approximately 5 × 10^8^ bacteria per mL, then centrifuged and washed in sterile 0.9% NaCl three times. The resulting leptospire pellets were resuspended in sterile water, subjected to repeated freeze–thaw cycles between −80 °C and 37 °C, and sonicated for 30 min on ice. To eliminate contaminating protein and nucleic acids, treatments with Proteinase K (200 μg/mL), DNase I (100 μg/mL) and RNase A (50 μg/mL) were performed prior to the extraction. We further purified the LPS in the aqueous phase since the majority of LPS was in this phase. To retrieve the leptospiral LPS from the aqueous phase, 90% phenol was used twice and extensively dialyzed against ddH_2_O to remove the phenol. The combined aqueous layers were extracted again with phenol. L06vLPS was precipitated by adding six volumes of 100% ethanol, and the samples were stored at −20 °C overnight, followed by centrifugation (8000*g*, 4 °C, 25 min) to obtain the crude L06vLPS.^29^ The protein was removed from the LPS using deoxycholate,^30^ and the sample was lyophilized to obtain the purified and dried L06vLPS. The purified L06vLPS was dissolved in sterile double-distilled water to obtain 1 mg/mL stock solution for the SDS-PAGE and biological activity analysis. The absence of protein contamination was verified using a BCA Protein Assay Kit (Pierce, Thermo Scientific). The molecular weight of L06vLPS was analyzed by SDS-PAGE, and the gel was stained with a Pro-Q Emerald 300 LPS Gel Stain Kit (MolecularProbes, Invitrogen Corp., Carlsbad, CA, USA). The Pro-Q Emerald 300 stain enables specific visualization of LPS. The polysaccharide component of LPS underwent periodate oxidation, after which the Pro-Q Emerald 300 dye reacted with the periodate-oxidized carbohydrate groups and created a bright green fluorescent signal.

### Chemical composition analysis of L06vLPS

Neutral monosaccharides were identified by PC-HPLC (pre-column high-performance liquid chromatography). The stock standards of glucose (Glc), mannose (Man), glucose acid (GlcA), galactose acid (GalA), glucosamine (GlcN), rhamnose (Rha), galactose (Gal), xylose (Xyl), galactosamine (GalN), arabinose (Ara) and fucose (Fuc) were accurately weighed and mixed in equal molar ratios. A dried L06vLPS sample (10 mg) and the standards were hydrolyzed separately and simultaneously with 2 mol/L trifluoroacetic acid (TFA) at 110 °C for 4 h, followed by a derivatization reaction with 3-methyl-1-phenyl-5-pyrazolone (PMP) at 70 °C for 1 h. The sample was then extracted with chloroform and analyzed by HPLC (Dionex UltiMate 3000, Sunnyvale, CA, USA). The method was optimized at a flow rate of 0.8 mL/min, an injection volume of 20 μL, and a temperature of 30 °C, and the signals were detected using a diode-array detector (245 nm). Separation was carried out over a C_18_ column with 4.6 mm diameter, 150 mm length and 5 μm particle diameter (Agilent ZORBAX Eclipse XDB-C18, Palo Alto, CA, USA) with a mixture of 0.1 mol/L phosphate buffer and acetonitrile as the mobile phase. The chromatographic peaks were identified by comparison of the retention time for standards and samples.

### Macrophage preparation and stimulation with LPS

BMDMs from C57BL/6 WT, TLR4^−/−^ and TLR2^−/−^ mice were obtained as previously described.^31^ Briefly, bone marrow was resuspended in ACK Lysing Buffer (Gibco, Thermo Scientific) and was subsequently washed. The myeloid precursors from the mouse bone marrow were cultured in Petri dishes (Nunc, Thermo Scientific) with modified Dulbecco’s modified Eagle’s medium containing 10% (v/v) heat-inactivated fetal bovine serum, 2 mM L-glutamine, 1 mM sodium pyruvate, 10 mM HEPES buffer and 30% L929 cell-conditioned medium at 37 °C and 5% CO_2_. After 5–6 days, mature BMDMs were digested with 5 mM EDTA and seeded into 96-well plates (1 × 10^5^ cells per mL) for LPS stimulation. To evaluate the activity of L06vLPS, BMDMs of WT mice were stimulated with 1, 10, 100, 1000 and 10 000 ng/mL of L06vLPS or 100 ng/mL *E. coli* LPS for 16 h.^32^ Then, BMDMs from WT, TLR4^−/−^, or TLR2^−/−^ mice were stimulated with *L. interrogans* strain 56606v (MOI=50), L06vLPS (10 000 ng/mL), *E. coli* LPS (10 ng/mL) or Pam3CSK4 (300 ng/mL) for 16 h. The potential of L06vLPS to antagonize *E. coli* LPS activation was also examined in the BMDMs. *E. coli* LPS (100 ng/mL) with or without the addition of L06vLPS (1, 10, 100, 1000 or 10 000 ng/mL) was added to WT BMDMs for 16 h. Tumor necrosis factor-alpha (TNF-α) concentrations from the cell culture supernatants were measured with an enzyme-linked immunosorbent assay (ELISA) kit for TNF-α (R&D Systems, San Francisco, CA, USA) according to the manufacturer’s protocols.

Peritoneal macrophages (PEMs) were harvested from WT, TLR4^−/−^ and TLR2^−/−^ mice. PEMs were elicited by injection of 1 mL of sterile 5% thioglycollate broth intraperitoneally as previously described.^20^ Five days later, PEMs were collected by peritoneal lavage with 5 mL of sterile PBS. The cells were resuspended in Dulbecco’s modified Eagle’s medium containing 10% fetal bovine serum, 2 mM L-glutamine, 1 mM sodium pyruvate, and 10 mM HEPES buffer and seeded in 96-well plates (1 × 10^5^ cells per mL). After 2 h of incubation, the cells were washed with PBS thoroughly to remove nonadherent cells. The PEMs were then stimulated with *L. interrogans* strain 56606v (MOI=50), L06vLPS (10000 ng/mL), *E. coli* LPS (10 ng/mL) or Pam3CSK4 (300 ng/mL) for 18 h, and the supernatants were tested for IL-6 according to the manufacturer’s protocols (R&D Systems, San Francisco, CA, USA).

### Reverse transcription and real-time PCR analysis

Total RNA was extracted from 250 μL of murine blood in 750 μL TRIzol LS Reagent (Thermo Scientific). Murine tissues were ground with a pestle in liquid nitrogen, and total RNA was extracted with TriPure Isolation Reagent (Roche Diagnostics GmbH, Mannheim, Germany). All RNA samples were treated with DNase I. RNA concentration was determined using a NanoDrop 2000 (Thermo Scientific). RNA (500 ng) was used for the generation of cDNA with a SuperScript III First-Strand Synthesis Super Mix Kit (Thermo Scientific). A PCR amplification reaction was performed with the ABI 7500 Fast Real-Time PCR System (Applied Biosystems). The PCR conditions were as follows: initial denaturation at 95 °C for 10 min, followed by 40 cycles of amplification with denaturation at 95 °C for 15 s and annealing temperature at 60 °C for 1 min. Primers for inducible nitric oxide synthase (iNOS), TNF-α, interleukin (IL)-6, IL-1β, glyceraldehyde-3-phosphate dehydrogenase (GAPDH) and 16S rRNA were as follows: iNOS, 5′-ACA TCG ACC CGT CCA CAG TAT-3′ and 5′-CAG AGG GGT AGG CTT GTC TC-3′ TNF-α, 5′-CCT GTA GCC CAC GTC GTA G-3′ and 5′-GGG AGT AGA CAA GGT ACA ACC C-3′ IL-6, 5′-TAG TCC TTC CTA CCC CAA TTT CC-3′ and 5′-TTG GTC CTT AGC CAC TCC TTC-3′ IL-1β, 5′-GAA ATG CCA CCT TTT GAC AGT G-3′ and 5′-TGG ATG CTC TCA TCA GGA CAG-3′ GAPDH, 5′-AGG TCG GTG TGA ACG GAT TTG-3′ and 5′-TGT AGA CCA TGT AGT TGA GGT CA-3′ 16S rRNA: 5′-AGC ACG TGT GTT GCC CTA GAC ATA-3′ and 5′-GTT GCC ATC ATT CAG TTG GGC ACT-3′. We calculated the efficiency of PCR amplification via standard curves using diluted samples as previously described.^33^ The RNA sample used was extracted from mouse peritoneal macrophages after *E. coli* LPS stimulation. The amplification efficiency of different genes ranged from ~90% to 101%. Fold changes in gene expression were calculated by 2^−ΔCt^ relative to GAPDH as the reference gene. All real-time PCR results were checked for specificity by melting-curve analysis as previously described.^25^

### Serum biochemical analysis

Whole blood, collected through the submandibular venous plexus, was centrifuged (3000 r/min, 5 min), and the serum was stored at −80 °C. The concentrations of serum bilirubin, aspartate transaminase (AST), blood urea nitrogen (BUN) and serum creatinine were measured using a UniCel DxC 800 Synchron autoanalyzer (Beckman Coulter, Brea, CA, USA).

### Statistical analysis

Statistical analysis was performed as described in each figure legend using GraphPad Prism 6.0 software (La Jolla, CA, USA). A two-tailed *t*-test was used for pairwise comparisons between groups. Linear regression was adopted to test for association between variables using Spearman’s rank correlation test. Data are presented as the mean±standard deviation (sd) and are considered significant when the *P* value is <0.05. All experiments were performed at least three times.

## RESULTS

### Acute leptospirosis with jaundice, subcutaneous and pulmonary hemorrhage in WT C57BL/6 mice

**Figure 1:**
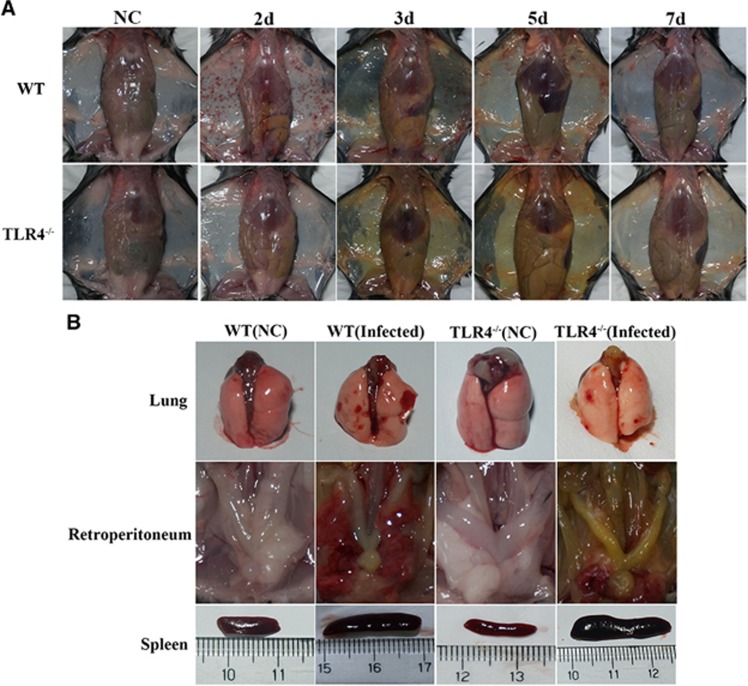
Gross examination of WT and TLR4^−/−^ mice infected with *L. interrogans* strain 56606v. WT and TLR4^−/−^ mice were infected with 2 × 10^8^ *L. interrogans* strain 56606v. (**A**) Gross observation of jaundice and hemorrhage in abdominal and subcutaneous tissue at 2, 3, 5 and 7 dpi. (**B**) Lungs, peritoneum and spleen from infected WT mice and TLR4^−/−^ mice at 3 dpi. NC: WT and TLR4^−/−^ mice inoculated with PBS were used as negative controls. Toll-like receptor, TLR; wild type, WT.

Ten-week-old C57BL/6 WT mice were infected by intraperitoneal injection of 2 × 10^8^ cells of *L. interrogans* serovar Autumnalis strain 56606v. Infected mice developed clinical signs, including rough hair, lethargy and reluctance to move, within 3 days postinfection (dpi). All mice developed subcutaneous and pulmonary hemorrhage, obvious jaundice and massive splenic enlargement (Figure 1).

To observe the distribution of leptospires in the blood of infected mice, blood samples were collected and leptospiral DNA was purified from the blood. The number of *Leptospira* 16S rRNA gene copies was determined by qPCR. At 8 h postinfection (hpi), leptospires were detected in the blood at approximately 6.8 × 10^4^ leptospires per μL. Bacteremia peaked at 2 dpi (2.5 × 10^5^ leptospires per μL blood) and then quickly decreased, and leptospires were eradicated at 14 dpi (Supplementary Figure S1).

**Figure 2:**
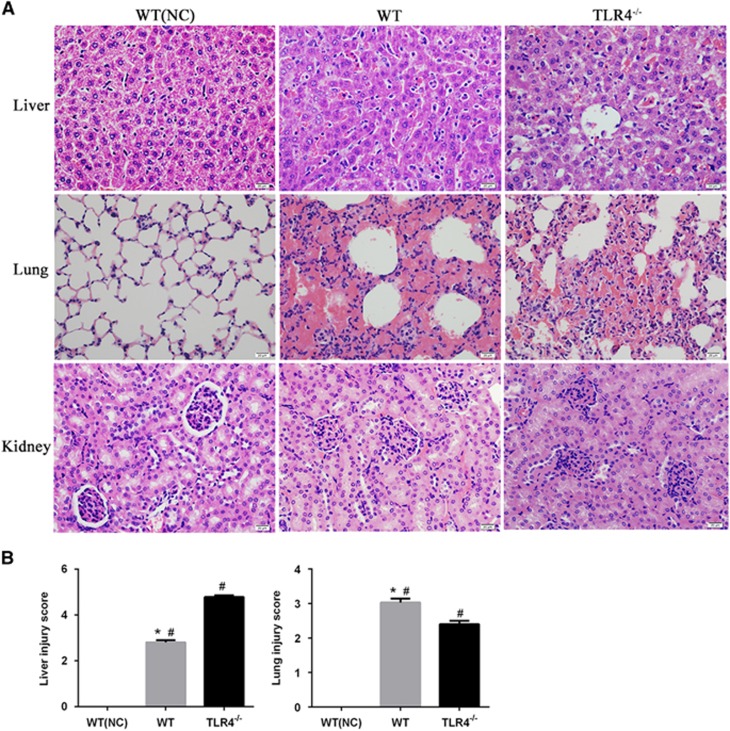
Histopathological lesions of WT and TLR4^−/−^ mice infected with *L. interrogans* strain 56606v. WT mice and TLR4^−/−^ mice were infected with 2 × 10^8^ cells of *L. interrogans* strain 56606v. (**A**) HE staining of liver, lungs and kidneys of control and infected WT and infected TLR4^−/−^ mice at 3 dpi. (Magnification, × 400). (**B**) Liver and lung injury scores in WT and TLR4^−/−^ mice were evaluated at 3 dpi. The data represent the mean±sd (*n*=3/group) and are representative of three independent experiments. *P<0.05 vs. TLR4^−/−^ mice and #*P*<0.05 vs. WT (NC) mice, as determined by a two-tailed *t*-test. Days postinfection, dpi; hematoxylin and eosin, HE; wild type, WT.

Histopathological changes were observed in the infected mice at 3 dpi. There was marked intra-alveolar hemorrhage in the lungs with slight-to-moderate inflammatory infiltrates and edema of the intra-alveolar septa (Figure 2). There was an evident loss in liver architecture, along with hepatocyte focal necrosis, Kupffer cell hypertrophy and hyperplasia (Figure 2). Severe lesions were not observed in kidney tissues (Figure 2). None of these features was observed in PBS-inoculated control animals (Figure 2).

**Figure 3:**
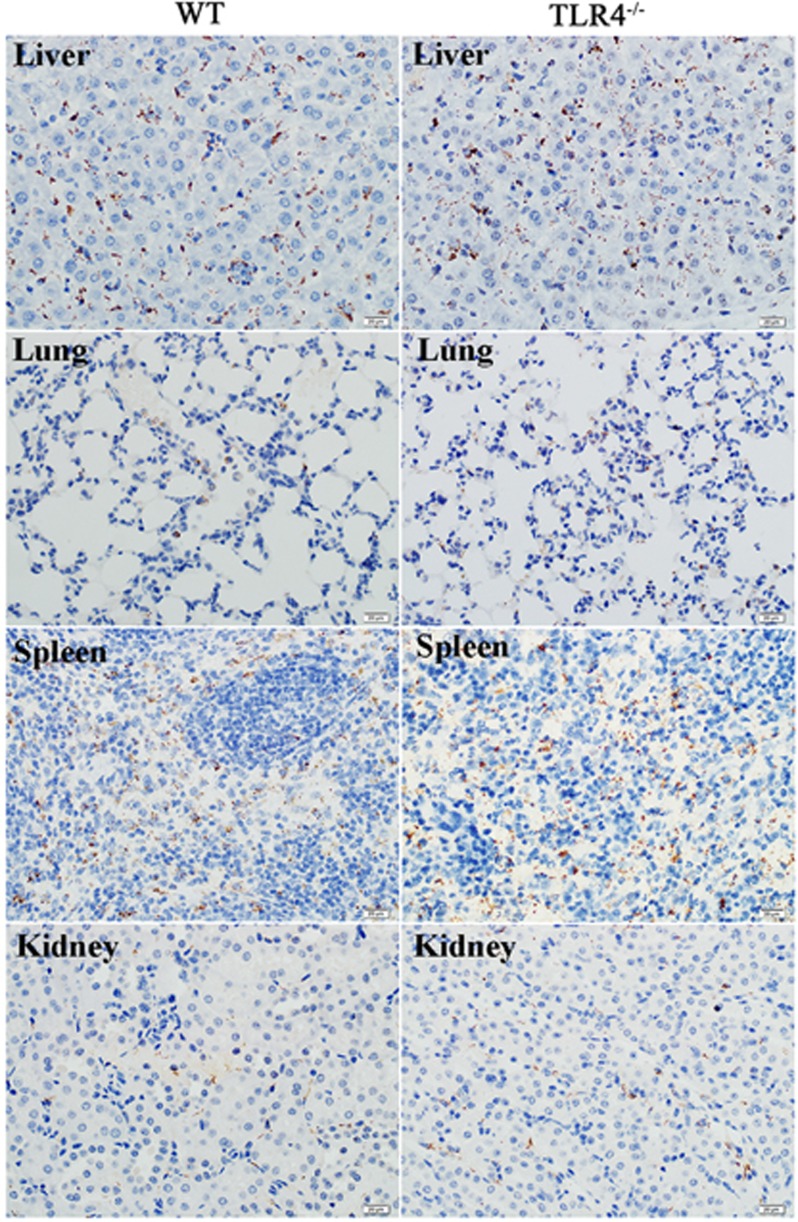
*L. interrogans* burden in various tissues of WT and TLR4^−/−^ mice infected with *L. interrogans* strain 56606v. Visualization of leptospires (brown threads and particles) in the liver, lungs, spleen and kidneys of WT and TLR4^−/−^ mice infected with *L. interrogans* strain 56606v at 2 dpi. Leptospires were stained by immunohistochemistry with antiserum specific for *L. interrogans* strain 56606v. (EnVision, magnification, × 400). Wild type, WT.

The dissemination of leptospires was observed in the lungs, liver, spleen and kidneys by immunohistochemistry as early as 2 dpi (Figure 3). The leptospiral burden then increased at 3 dpi and diminished at 5 dpi (Supplementary Figure S2).

### LPS of *L. interrogans* strain 56606v signals via TLR4 and not TLR2 in murine BMDMs and PEMs

**Figure 4:**
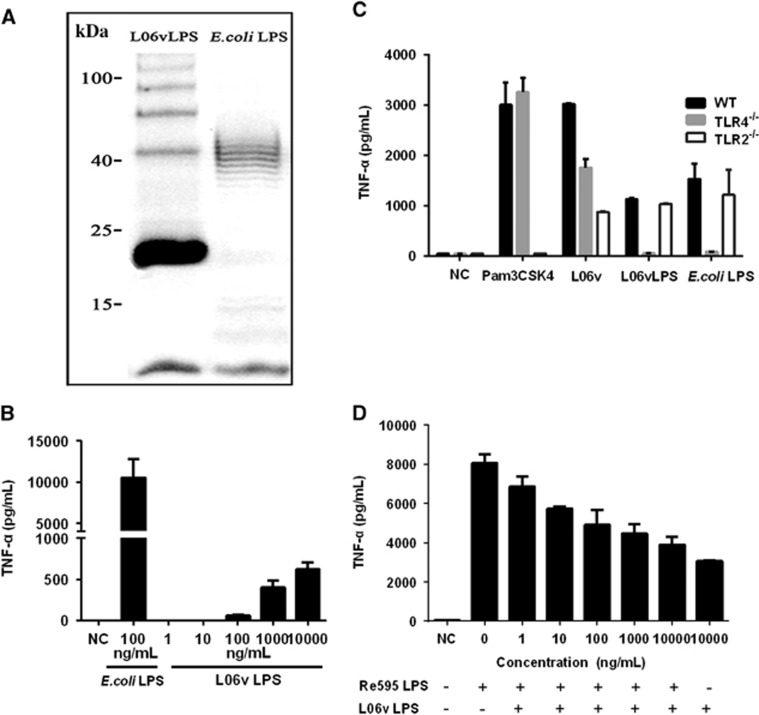
Electrophoretic profile of LPS extracted from *L. interrogans* strain 56606v; L06vLPS-induced TNF-α production in BMDMs of WT, TLR4^−/−^ and TLR2^−/−^ mice. (**A**) Lipopolysaccharides from *L. interrogans* strain 56606v (L06vLPS) (10 μg) and *E. coli* O55:B5 (*E. coli* LPS; 1 μg) were analyzed using 12% SDS-PAGE and Pro-Q Emerald 300 staining. The positions of molecular mass markers (in kilodaltons) are indicated on the left. (**B**) BMDMs of WT mice were stimulated with PBS (NC), *E. coli* LPS or increasing doses of L06vLPS for 16 h. (**C**) BMDMs of WT, TLR4^−/−^, or TLR2^−/−^ mice were stimulated with PBS (NC), *L. interrogans* strain 56606v (L06v, MOI=50), L06vLPS (10 000 ng/mL), *E. coli* LPS (10 ng/mL) or Pam3CSK4 (300 ng/mL) for 16 h. (**D**) BMDMs of WT mice were stimulated with PBS (NC) or with *E. coli* LPS (100 ng/mL) and the addition of L06vLPS (0, 1, 10, 100, 1000 or 10 000 ng/mL) for 16 h. TNF-α concentrations from cell culture supernatants were measured by ELISA. Data are expressed as the mean±sd of triplicate samples from one experiment and are representative of at least three independent experiments. Lipopolysaccharide, LPS; phosphate-buffered saline, PBS; Toll-like receptor, TLR; tumor necrosis factor, TNF; wild type, WT.

To better characterize the role of LPS in the host-pathogen interactions of strain 56606v infection, we purified L06vLPS and studied its biological characteristics. SDS-PAGE analysis of L06vLPS showed a ‘typical’ ladder pattern of O-antigen (Figure 4A). The molecular weight of L06vLPS was appropriately 23 kDa (Figure 4A). PC-HPLC analysis of the carbohydrate composition of *L. interrogans* strain 56606v LPS showed that the monosaccharides present in L06vLPS included mannose (Man), glucosamine (GlcN), rhamnose (Rha), galactose acid (GalA), galactose (Gal), xylose (Xyl), arabinose (Ara) and Fucose (Fuc) (Supplementary Figure S3). To evaluate the activity of L06vLPS in stimulating mouse BMDMs, we constructed a dose-response curve using L06vLPS with a concentration range of 1 to 10 000 ng/mL. Consistent with previous studies, L06vLPS was much less potent than *E. coli* LPS in stimulating TNF-α expression by WT mouse BMDMs, and its threshold for inducing TNF-α production was 100 ng/mL (Figure 4B).

A previous study showed that TLR2 played a predominant role in recognizing *Leptospira* LPS in both mice and humans.^21^ To determine the pattern of L06vLPS recognition by TLRs, we stimulated the BMDMs of WT, TLR4^−/−^, or TLR2^−/−^ mice with *L. interrogans* strain 56606v, L06vLPS, *E. coli* LPS or Pam3CSK4, respectively, and tested TNF-α production in the supernatants at 16 hpi. The results showed that TNF-α production to whole leptospires decreased in both TLR4^−/−^ and TLR2^−/−^ mice (Figure 4C). Unexpectedly, production of TNF-α to L06vLPS in WT mouse BMDMs was similar to that in TLR2^−/−^ mouse BMDMs; however, the production of TNF-α was almost undetected in TLR4^−/−^ mouse BMDMs (Figure 4C). Similar results were obtained when PEMs were stimulated (Supplementary Figure S4). The production of IL-6 was similar in TLR2^−/−^ and WT mouse PEMs but was almost undetected in TLR4^−/−^ mouse PEMs. These results demonstrated that *Leptospira* LPS acts primarily on TLR4, not TLR2, to mediate TNF-α production, which is inconsistent with previous findings.^20, 21^ To determine whether L06vLPS has the same binding sites of murine TLR4 complex as *E. coli* LPS, the potential of L06vLPS to antagonize *E. coli* LPS activation was examined in BMDMs. The result showed that L06vLPS antagonizes *E. coli* LPS in a dose-dependent manner as determined by TNF-α production by BMDMs (Figure 4D).

### TLR4^−/−^ mice present with more severe jaundice but milder pulmonary and subcutaneous hemorrhages along with higher bacterial load compared with WT mice upon infection with *L. interrogans* strain 56606v

**Figure 5:**
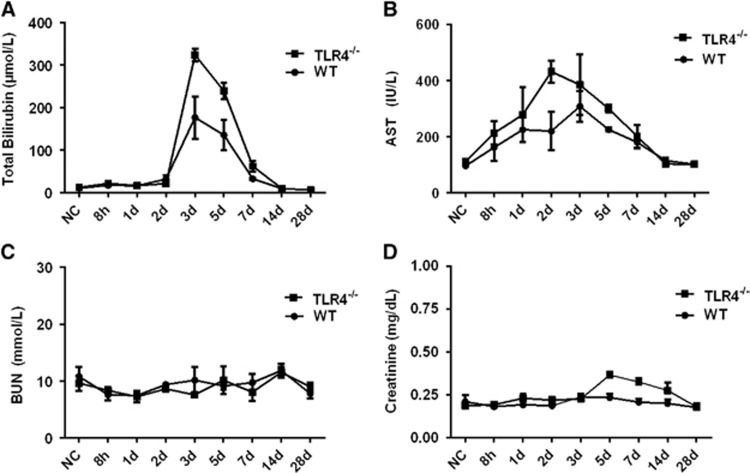
Serological markers for liver and kidney damage in WT and TLR4^−/−^ mice infected with *L. interrogans* strain 56606v. WT or TLR4^−/−^ mice were infected by intraperitoneal injection of 2 × 10^8^ bacteria/mouse. The levels of serum bilirubin (**A**), aspartate transaminase (AST) (**B**), blood urea nitrogen (BUN) (**C**) and serum creatinine (**D**) were measured using a UniCel DxC 800 Synchron autoanalyzer at 8 h, 1, 2, 3, 5, 7, 14 and 28 dpi. The results are from three animals per time point and are representative of three independent experiments.

**Figure 6:**
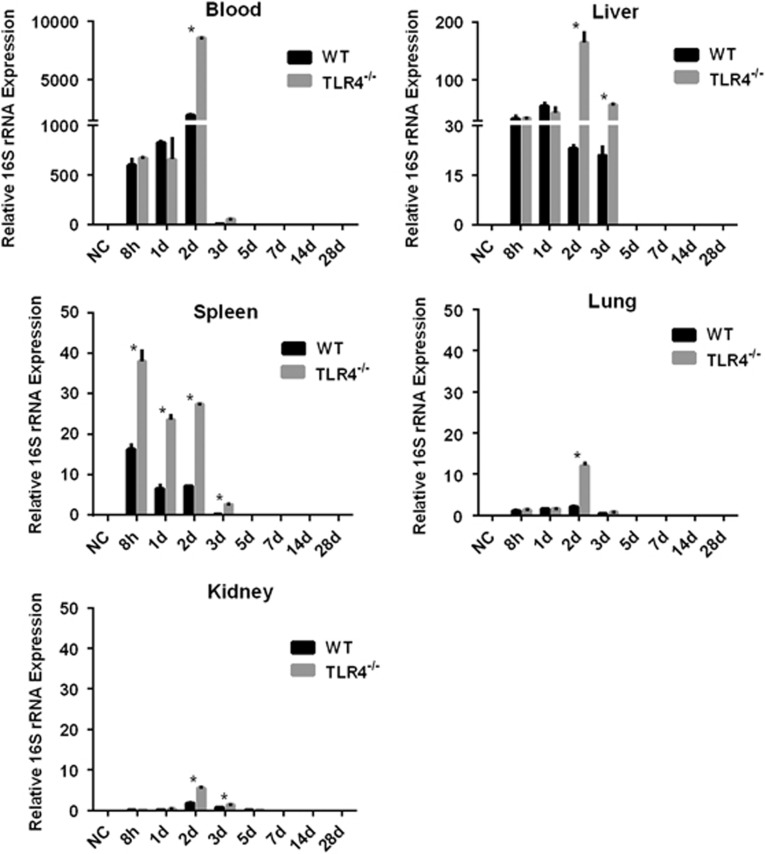
The live *Leptospira* load in the blood and tissues of WT and TLR4^−/−^ mice infected with *L. interrogans* strain 56606v. WT or TLR4^−/−^ mice were infected by intraperitoneal injection of 2 × 10^8^ bacteria per mouse. The kinetics of the leptospiral burden in blood and tissues from infected WT and TLR4^−/−^ mice were measured by the ratio of *Leptospira* 16S rRNA to the murine *GAPDH* gene at 8 h, 1, 2, 3, 5, 7, 14 and 28 dpi. Data represent mean±sd of the levels of leptospires in blood. The results are from three animals per time point and are representative of three independent experiments. *P<0.05 between WT and TLR4^−/−^ mice. Wild type, WT.

To further understand the contribution of murine TLR4 in the pathogenesis of leptospirosis, adult (10 weeks) WT and TLR4^−/−^ mice were infected via the ip route with 2 × 10^8^ cells of *L. interrogans* strain 56606v, and the pathological lesions were examined at 8 h, 1, 2, 3, 5, 7, 14 and 28 dpi. The infected WT and TLR4^−/−^ mice all had obvious jaundice along with pulmonary, subcutaneous and retroperitoneal hemorrhages and massive splenic enlargement (Figure 1). None of the WT or TLR4^−/−^ mice died from the infection during the course of the study. Strikingly, TLR4^−/−^ mice had milder pulmonary, subcutaneous and retroperitoneal hemorrhages but more severe jaundice than WT mice (Figure 1). HE staining also revealed pulmonary hemorrhage and mild-to-moderate inflammatory infiltration and edema of the intra-alveolar septa in TLR4^−/−^ and WT mice (Figure 2 and Supplementary Table S1). Histological findings in the liver showed inflammation in the livers of all infected mice, but architecture loss and focal necrosis of hepatocytes were more severe in TLR4^−/−^ than in WT mice (Figure 2 and Supplementary Table S1). Severe lesions were not observed in the kidney tissues of TLR4^−/−^ or WT mice (Figure 2), while BUN and serum creatinine remained at normal levels in both TLR4^−/−^ and WT mice (Figure 5). The live *Leptospira* loads in the blood and tissues were measured by the ratio of *Leptospira* 16S rRNA to the murine *GAPDH* gene. Leptospires multiplied in the blood, liver, spleen, lungs and kidneys at 8 h, then peaked at 2 dpi, and few or no bacteria were detected at 5 dpi in WT and TLR4^−/−^ mice. At 2 dpi, the *Leptospira* load was significantly higher in the blood, liver, spleen, lungs and kidneys in infected TLR4^−/−^ than WT mice (Figure 6). Immunohistochemical studies using an antibody against *L. interrogans* strain 56606v confirmed the presence of leptospires in different organs. Moreover, a significantly higher *Leptospira* burden was observed at 2 dpi in the liver, lungs, spleen and kidneys of TLR4^−/−^ than in those of WT mice (Figure 3). Two liver serological markers, total bilirubin and AST, were found elevated at 3 and 5 dpi in infected mice and were significantly higher in TLR4^−/−^ than in WT mice (Figure 5).

### Defective expression of proinflammatory cytokines and iNOS in *Leptospira*-infected TLR4^−/−^ mice

**Figure 7:**
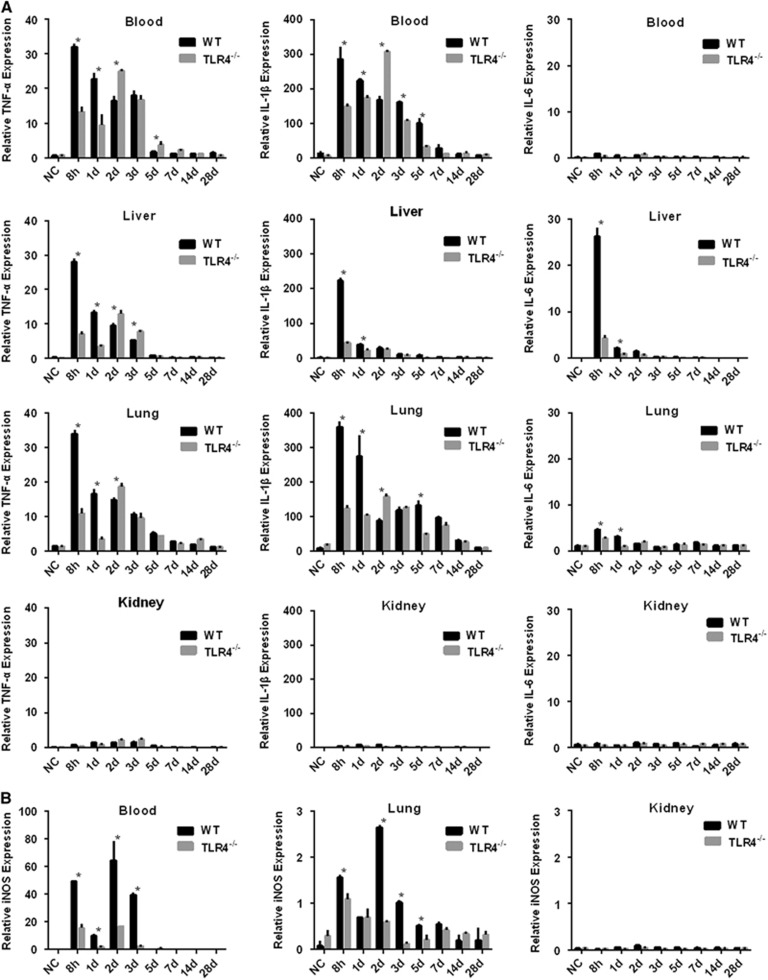
Levels of cytokines and iNOS in blood and tissues of WT and TLR4^−/−^ mice infected with *L. interrogans* strain 56606v. Blood and tissues were collected after inoculation with 2 × 10^8^ leptospires per mouse at different time points. For real-time PCR, the results are normalized to the *GAPDH* gene as an internal control. (**A**) The relative mRNA expression levels of proinflammatory cytokines including TNF-α, IL-1β and IL-6 were quantified by real-time PCR in the blood, liver, lungs and kidneys of infected WT or TLR4^−/−^ mice. (**B**) Relative mRNA expression levels of iNOS quantified by real-time PCR in the blood and lungs of infected WT or TLR4^−/−^ mice. Data are mean±sd from duplicate measurements of three animals per time point and are representative of three independent experiments. *P<0.05 between WT and TLR4^−/−^ mice. Interleukin, IL; inducible nitric oxide synthase, iNOS; Toll-like receptor, TLR; tumor necrosis factor, TNF; wild type, WT.

To better understand the roles of TLR4 in the induction of the inflammatory response caused by *L. interrogans* strain 56606v, the levels of certain cytokine mRNAs in the blood, liver, kidneys and lungs from WT and TLR4^−/−^ mice were measured at determined time points. Soon after infection (8 hpi), the mRNA levels of the proinflammatory cytokines TNF-α and IL-1β were significantly elevated in the blood of WT and TLR4^−/−^ mice. The mRNA levels of TNF-α and IL-1β peaked at 8 hpi and then decreased in the following days in WT mice, whereas they rose gradually and peaked at 2 dpi in TLR4^−/−^ mice (Figure 7A). At 2 dpi, the higher levels of the cytokine mRNAs in the blood of TLR4^−/−^ mice may be related to the higher leptospiral load in the TLR4^−/−^ mice (Figure 6). In the liver, TNF-α, IL-1β and IL-6 were highly induced in WT mice as soon as at 8 hpi and then decreased rapidly, while the cytokine levels of TLR4^−/−^ mice were relatively lower than those of WT mice (except for TNF-α at the time points of 2 and 3 dpi; Figure 7A). In the lungs, the expression profiles of proinflammatory cytokines were similar to those in the blood. The expression of TNF-α and IL-1β was higher in WT mice than TLR4^−/−^ mice at 8 hpi and 1 dpi, while at 2 dpi the expression of cytokines in TLR4^−/−^ mice increased rapidly and surpassed the level in WT mice (Figure 7A). As with proinflammatory cytokines, the level of iNOS mRNA was elevated in the blood and tissues of infected mice after 8 hpi. In WT mice, it peaked at 2 dpi and was markedly higher in the blood and lungs than in TLR4^−/−^ mice (Figure 7B). The expression of all three cytokines and iNOS remained at basal levels in the kidneys of WT mice and TLR4^−/−^ mice throughout the experiment.

## DISCUSSION

In contrast to guinea pigs and hamsters, which have been successfully used as animal models of acute leptospirosis, mice are relatively resistant to leptospiral infection.^7, 8, 10^ Acute leptospirosis develops primarily in mice younger than 6 weeks of age or in immunocompromised mice with certain immune response-related genes deleted.^12, 13^ Some reports of adult mice infected with leptospires indicated that they had no clinical signs and exhibited renal colonization without histopathological changes;^16^ other reports showed chronic infection with kidney lesions and fibrosis.^17, 34^ However, signs of severe acute leptospirosis induced in immunocompetent mice were recently reported by Ratet *et al*,^18^ who showed that C57BL/6 mice (7–10 weeks old) infected with *L. interrogans* serovar Manilae died within a few days, with clinical manifestations of septicemia and renal colonization.^18^ In this study, we analyzed the outcome of 10-week-old C57BL/6 mice (WT) challenged with *L. interrogans* serovar Autumnalis strain 56606v, which showed bloodstream and tissue dissemination of leptospires and typical leptospirosis symptoms including jaundice and pulmonary hemorrhage. *Leptospira* RNA in the blood and tissues (including the kidneys) were cleared at 5 dpi, indicating the absence of chronic infection. Meanwhile, *Leptospira* DNA could still be detected at 7 dpi. This might be explained by the longer half-life of DNA. However, kidney involvement such as interstitial nephritis and kidney fibrosis was absent in our model. Higher *Leptospira* loads were detected in the blood and liver compared with the kidneys, suggesting that *L. interrogans* strain 56606v might be more inclined to cause septicemia and liver damage than renal disorder. This result was different from previous studies reporting that *Leptospira* burden is prominent in the kidneys, while minimal colonization or inflammation is observed in the lungs and livers of mice infected with *L. interrogans* serovar Copenhageni^15, 35^ Some studies reported that leptospiral outer membrane proteins (OMPs) and lipoproteins play important roles in leptospiral kidney injury.^36, 37^ An *in vitro* experiment showed that OMPs and lipoproteins expressed during leptospiral infection seem to facilitate leptospiral attachment to kidneys.^37^ Moreover, leptospiral OMPs and lipoproteins were reported to provoke a robust proinflammatory response in renal tubule cells and thereby to play a key role in the pathogenesis of renal disease in leptospirosis.^38, 39^ Our previous research using comparative genomic hybridization analysis showed that LipL36 and some OMPs were absent in *L. interrogans* strain 56606v (Lin4) compared to *L. interrogans* serovar Copenhageni strain Fiocruz F1-130.^40^ These absent OMPs may contribute to the colonization of leptospires in renal tubule cells and cause tubulointerstitial nephritis. The probable pathogenic functions of these OMPs merit further exploration.

This model of sublethal *Leptospira interrogans* infection, with typical clinical manifestations of jaundice and hemorrhage, is novel and could be used to investigate the pathogenesis of pulmonary hemorrhage and liver damage in severe leptospirosis.

To ascertain whether other virulent leptospires can cause similar leptospirosis symptoms in C57BL/6 mice, we also infected C57BL/6 WT mice with the same dose and via the same route of inoculation with *L. interrogans* serovar Lai, which can cause typical acute leptospirosis with jaundice and pulmonary hemorrhage in guinea pigs.^8^ *L. interrogans* strain Lai did not cause any obvious lesions in C57BL/6 WT mice up to 7 dpi, and this indicated that *L. interrogans* strain 56606v induced typical acute leptospirosis symptoms in mice in a strain-specific manner.

LPS plays a very important role in host-pathogen interactions by triggering the activation of the immune system, which generally occurs via sensitizing the TLR4/MD2 complex.^41, 42^ However, not all Gram-negative bacteria LPS are recognized by the TLR4 complex. For instance, *Porphyromonas gingivalis* LPS and *Burkholderia pseudomallei* LPS were reported to be recognized by TLR2.^43, 44^ The differential TLR signaling pathways triggered by LPS might be explained by the three-dimensional conformation of lipid A.^45^ ‘Classical’ lipid A such as that in *E. coli* LPS is conical in shape and signals through TLR4 binding. However, atypical cylindrical lipid A, which has different numbers or symmetrical positions of acyl groups, a different disaccharide backbone, and different carboxyl or phosphate groups, may trigger cytokine production through TLR2.^45^ Elegant studies by Werts’ group showed that TLR2 played a predominant role in recognizing *Leptospira* LPS in mouse PEMs.^20^ Interestingly, we found in this study that purified L06vLPS signals mainly through TLR4 rather than TLR2 in murine BMDMs and PEMs. One likely explanation for this difference is that L06vLPS is different in structure from other *Leptospira* strains, leading to alterations of the classic TLR2-dependent PAMP-PRR recognition. The *rfb* locus of *L. interrogans* serovar Autumnalis strain 56606v (Lin4) has been sequenced and identified (GenBank: FJ976887.1).^19^ Genes involved in the biosynthesis of the O-antigen within the *rfb* locus of *L. interrogans* serovar Autumnalis were compared with those of *L. interrogans* serovar Copenhageni and serovar Lai (Supplementary Figure S5). The comparison revealed that a sequence of ~30 kb at the 3′ end of *rfb* locus and a sequence of ~19 kb at the 5′ end of *rfb* locus in strain 56606v are almost identical to their counterparts in serovar Copenhageni and serovar Lai (Supplementary Figure S5). O-antigen biosynthesis genes in the middle of the *rfb* locus were specific for *L. interrogans* strain 56606v, which may contribute to serological and TLR recognition differences in LPS among the *Leptospira spp*. Another explanation is that different reservoirs of *Leptospira spp*. may change the recognition of LPS by TLR. The natural reservoir of *L. interrogans* serogroup Icterohaemorrhagiae serovar Lai mainly includes the families Muridae and Soricidae, followed by swine, bovines and canines.^46^ Meanwhile, *L. interrogans* strain 56606 belongs to serogroup Autumnalis serovar Autumnalis, whose natural reservoir includes not only Muridae, swine, bovines and canines^46^ but also amphibians, such as toads, *Hylarana taipehensis*, and *Enhydris chinensis*.^46^ *Yersinia pestis* produces hexaacyl LPS when growing in fleas, while tetraacyl LPS production occurs in mammalian hosts.^47^ Changes in leptospiral LPS have also been reported in different models of infection.^48^ Different host-pathogen interactions may influence *Leptospira* LPS; these may have different biological properties. Moreover, *in vitro* vs *in vivo* culture of *Leptospira* may further influence the chemical properties of its LPS.^24, 49^ The difference in *Leptospira* LPS may also be due to the different phase (aqueous or phenol) in which it was purified. In this study, we found that the majority of *L. interrogans* strain 56606v LPS was in the aqueous phase, and thus, LPS was purified from the aqueous phase. In a previous study, LPS from strain Verdun was purified from the phenol phase.^21^ The saccharide residues of *Leptospira* LPS are different when purified from the aqueous phase as opposed to the phenol phase.^50^ As clone purification by agar plate was not routinely used in *Leptospira* culture, LPS variation is likely to occur in either *in vitro* culture or animal infection, which may influence the saccharide residues of *Leptospira* LPS and whether they localize in the aqueous or phenol phase.

In this study, the murine macrophages exhibited lower TNF-α production upon L06vLPS stimulation compared with *E. coli* LPS, which was consistent with the previous findings.^20^ Distinct structural features of *Leptospira* lipid A revealed by Reatz and Werts’s group may explain the reduced endotoxic potency.^22^ Another interesting finding in this study is that L06vLPS can potently antagonize the cytokine release of BMDMs stimulated by *E. coli* LPS. It seems that a high concentration of L06vLPS directly competes with *E. coli* LPS for binding sites on the murine TLR4 complex, acting as a weak agonist of TLR4. It is well known that LPS recognition by an animal host can initiate immune signaling pathways that are both damaging and protective. Production of weakly stimulatory LPS may be essential for leptospires to cause disease while stimulating minimal or negligible TLR4 signal responses, thereby avoiding containment by local immune response. The feature of antagonism of TLR4 is likely to be another important activity in facilitating *Leptospira* infection. When leptospires in contaminated water infect an animal host via cut or abraded skin, such antagonistic activity will cause multiple other LPS-containing pathogenic species in the wound to become inert with respect to TLR4 stimulation during early infection.

Considering that L06vLPS signals mainly through TLR4, we therefore infected TLR4^−/−^ mice with strain 56606v to explore the role of L06vLPS interaction with TLR4 in the pathogenesis of acute leptospirosis. Our results showed that TLR4^−/−^ mice had significantly higher numbers of *Leptospira* in the blood and tissues, which were consistent with a previous report that intact TLR4 signaling was important in the control of tissue leptospiral burden.^13, 14^ Moreover, TLR4^−/−^ mice presented more severe jaundice and higher bilirubin and AST levels compared with WT mice over the course of the disease, which was in accordance with the higher *Leptospira* burden and lesions in the liver of TLR4^−/−^ mice that peaked on 2 dpi and remained at a relatively high level until 3 dpi. The result was consistent with a previous study by Miyahara *et al*, who demonstrated that the mechanism of jaundice in leptospirosis is invasion of Disse’s space by the *Leptospira*, causing detachment of intercellular junctions between hepatocytes and destruction of bile canaliculi.^51^ In this study, there was markedly elevated expression of the cytokines TNF-α, IL-1β and IL-6 at the early stage of infection in the liver of WT mice compared with TLR4^−/−^ mice. Proinflammatory cytokines play an important role in initiating host immune response in the early stages of infection. Athanazio *et al* reported that TNF-α receptor-deficient mice exhibited more severe residual kidney lesions after *Leptospira* infection, suggesting that TNF-α may be important in preventing severe acute lesions.^52^ At 2 dpi, the levels of TNF-α mRNA in the blood and liver of TLR4^−/−^ mice were lower than those of WT mice. It was reported that *Leptospira* infection leads to significant TLR2- and TLR4-independent inflammatory responses.^14^ The higher leptospiral load in TLR4^−/−^ mice at 2 dpi may cause a high level of TLR4-independent inflammatory response. Thus, the severe liver injury in *Leptospira*-infected TLR4^−/−^ mice in this study was more likely due to the deficiency in proinflammatory cytokine production and, subsequently, the high burden of hepatic *Leptospira*.

Pulmonary hemorrhage is one of the typical clinical signs of leptospirosis.^1, 3^ However, the exact mechanism of bleeding remains to be elucidated. Here we report milder pulmonary and subcutaneous hemorrhage in TLR4^−/−^ mice than in WT mice, suggesting that TLR4 contributes, at least partially, to hemorrhage in leptospirosis. To further analyze the possible correlation between the severity of the hemorrhage and TLR4-related immune response, we evaluated the transcripts of TNF-α, IL-1β, IL-6 and iNOS in WT and TLR4^−/−^ mice. We observed markedly higher levels of TNF-α and IL-1β mRNA in the blood and lungs of WT mice compared to TLR4^−/−^ mice in early stages (except the time point at 2 dpi). Moreover, one compelling point raised by our study was that the severe subcutaneous and pulmonary hemorrhage caused by the pathogenic *Leptospira* seemed to be more related to the overexpression of proinflammatory mediators than to the high load of *Leptospira*, since TLR4^−/−^ mice showed a higher *Leptospira* load in the blood and lungs but milder hemorrhage than WT mice, whereas the significantly elevated serum and pulmonary levels of iNOS mRNA were observed in WT mice compared with TLR4^−/−^ mice, reaching a peak on 2 dpi, coinciding with the time point when pulmonary and subcutaneous hemorrhages were the most severe. Several studies have suggested that elevated iNOS expression may contribute to pulmonary hemorrhage in leptospirosis.^49, 53, 54^ iNOS is an important component of innate immunity, acting as a protective barrier against pathogens, mediating bacterial clearance and controlling infection,^55, 56^ while simultaneously contributing to cell and tissue injury.^14^ In this study, the defective expression of proinflammatory mediators by TLR4^−/−^ mice is probably responsible, to some extent, for the ineffective control of bacterial replication. Thus, we postulate that iNOS, together with other proinflammatory cytokines, plays an important role in mediating bacterial clearance by the innate immune system while also inducing host immunopathological tissue injury.

There are some published cases of severe leptospirosis in which corticosteroid administration improved survival among patients, while there is still insufficient evidence to suggest the effectiveness of immunosuppressive treatments in severe leptospirosis.^57, 58, 59^ In this study, we observed an alleviation of pulmonary hemorrhage in TLR4^−/−^ mice, possibly due to suppression of the ‘storm’ of proinflammatory cytokine production. This suggests the potential of combining TLR4 blockade and antibiotics in the treatment of severe leptospirosis with manifestations of pulmonary hemorrhage.

Indeed, in a previous study, no severe clinical symptoms were observed in WT mice after infection with *L. interrogans* serovar Copenhageni strain Fiocruz F1-130, whereas TLR4^−/−^ mice were more susceptible, developing lethal acute leptospirosis upon infection with the same dose.^14^ In the present study, no lethality was observed in either WT or TLR4^−/−^ mice after infection with strain 56606v. One likely explanation for this difference is that strain 56606v is not a highly virulent strain of *L. interrogans* and that TLR4^−/−^ mice could survive with high levels of bacteremia (>2.5 × 10^5^ leptospires per μL blood). Another possibility is that the TLR4-independent pathway, presumably TLR2 or other innate immune mechanisms, is responsible for host defense against leptospiral infection.

Overall, we established a murine model of self-resolving leptospirosis using adult, immunocompetent C57BL/6 mice, with typical clinical manifestations of human leptospirosis including jaundice and pulmonary hemorrhage. This is promising for future studies of leptospiral pathogenesis and host-bacteria interaction. Our data suggest a protective role of TLR4 in strain 56606v clearance, while the receptor is also associated with bleeding in acute leptospirosis, possibly through induction of downstream proinflammatory mediators. This might offer insight into future treatment regimens for patients with severe bleeding.
