# Supplementary material for: A new model of self-resolving leptospirosis in mice infected with a strain of Leptospira interrogans serovar Autumnalis harboring LPS signaling only through TLR4
Source: Emerg Microbes Infect. 2017 May 24;6(5):e36–. doi: 10.1038/emi.2017.16 (PMC5520481; doi:10.1038/emi.2017.16)
Supplement: Supplementary Figure S1 [file emi201716x1.docx]

**Supplementary Figure S1 Variation of *L. interrogans* burdens in the blood of C57BL/6 mice infected with *L. interrogans* strain 56606v.**


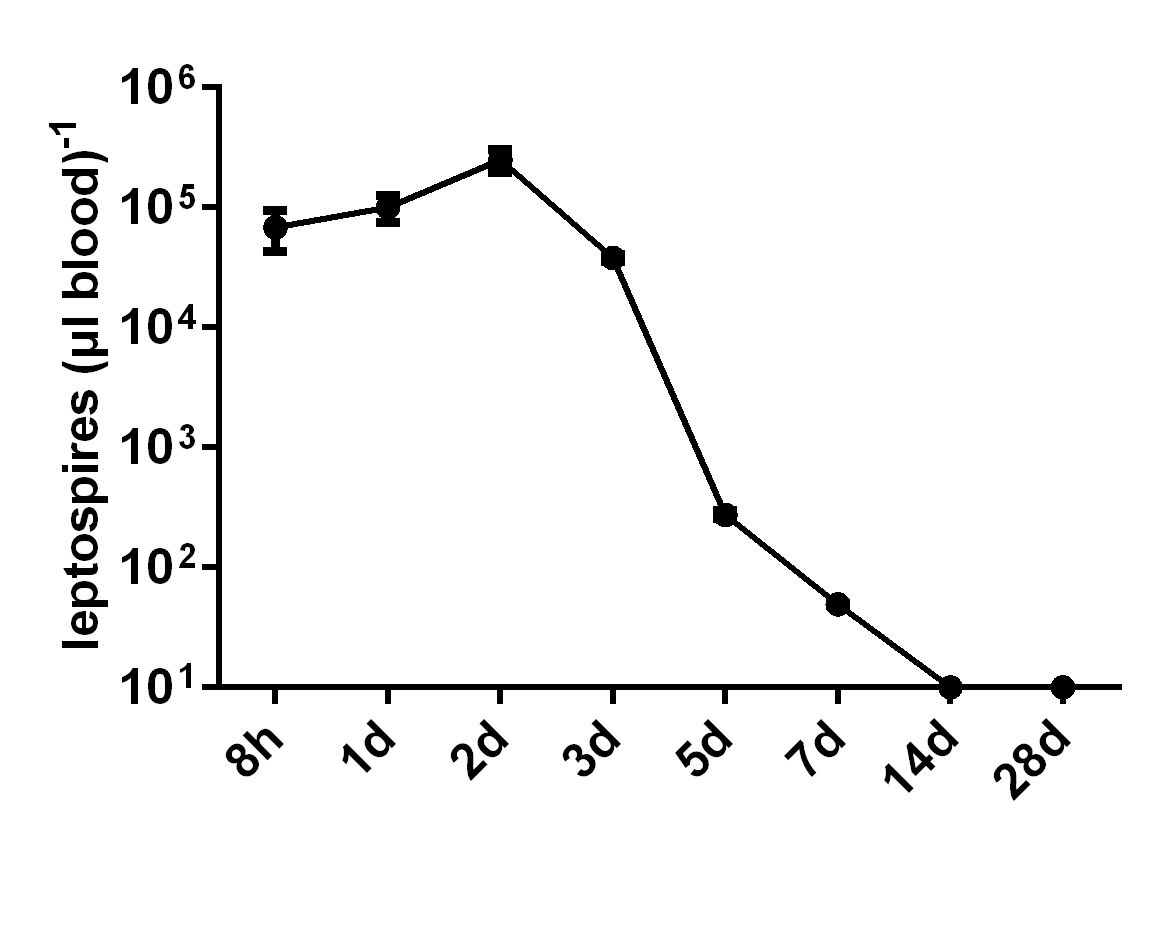


The leptospiral burden in the blood of infected C57BL/6 mice was analyzed by measuring copies of the 16S rRNA gene at 8 h, 1 d, 2 d, 3 d, 5 d, 7 d, 14 d and 28 dpi. A standard curve was generated with serial dilutions (10^1^ to 10^7^) of *L. interrogans* strain 56606v genomic DNA. Data represent mean ± SD of the leptospiral load in the blood. The results are from three animals per time point and are representative of three independent experiments.
