# Supplementary material for: A new model of self-resolving leptospirosis in mice infected with a strain of Leptospira interrogans serovar Autumnalis harboring LPS signaling only through TLR4
Source: Emerg Microbes Infect. 2017 May 24;6(5):e36–. doi: 10.1038/emi.2017.16 (PMC5520481; doi:10.1038/emi.2017.16)
Supplement: Supplementary Figure S2 [file emi201716x2.docx]

**Supplementary Figure S2 *L. interrogans* burden in various tissues of C57BL/6 mice infected with *L. interrogans* strain 56606v.**


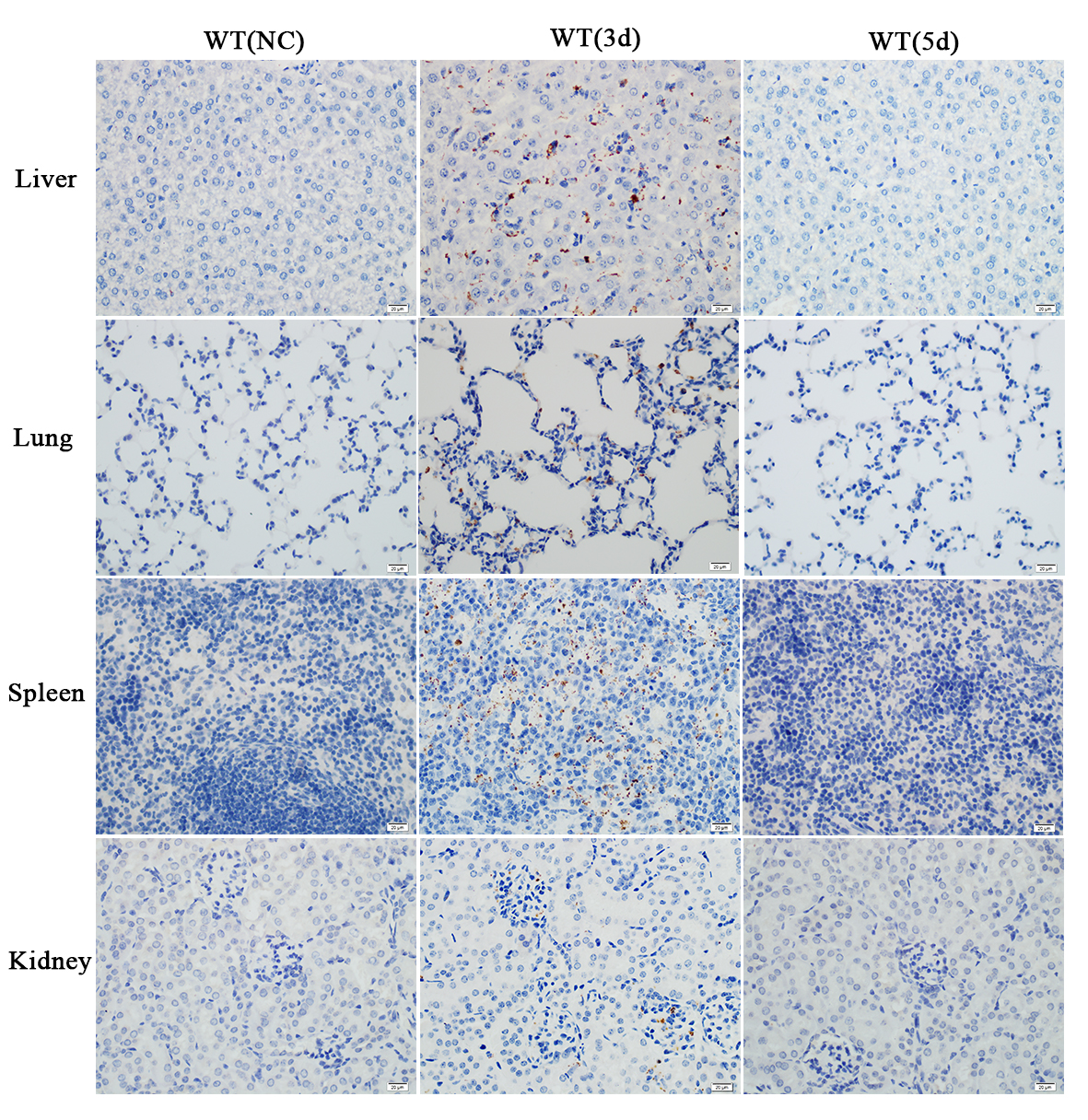


Visualization of leptospires (brown threads and particles) in the liver, lungs, spleen and kidneys of C57BL/6 control and C57BL/6 mice infected with *L. interrogans* strain 56606v at 3 and 5 dpi. Leptospires were stained by immunohistochemistry with antiserum specific for *L. interrogans* strain 56606v. (EnVision, magnification, × 400.)
