# Supplementary material for: A new model of self-resolving leptospirosis in mice infected with a strain of Leptospira interrogans serovar Autumnalis harboring LPS signaling only through TLR4
Source: Emerg Microbes Infect. 2017 May 24;6(5):e36–. doi: 10.1038/emi.2017.16 (PMC5520481; doi:10.1038/emi.2017.16)
Supplement: Supplementary Figure S3 [file emi201716x3.docx]

**Supplementary Figure S3 Monosaccharide composition analysis of LPS of *L. interrogans* serovar Autumnalis strain 56606v by PC-HPLC.**


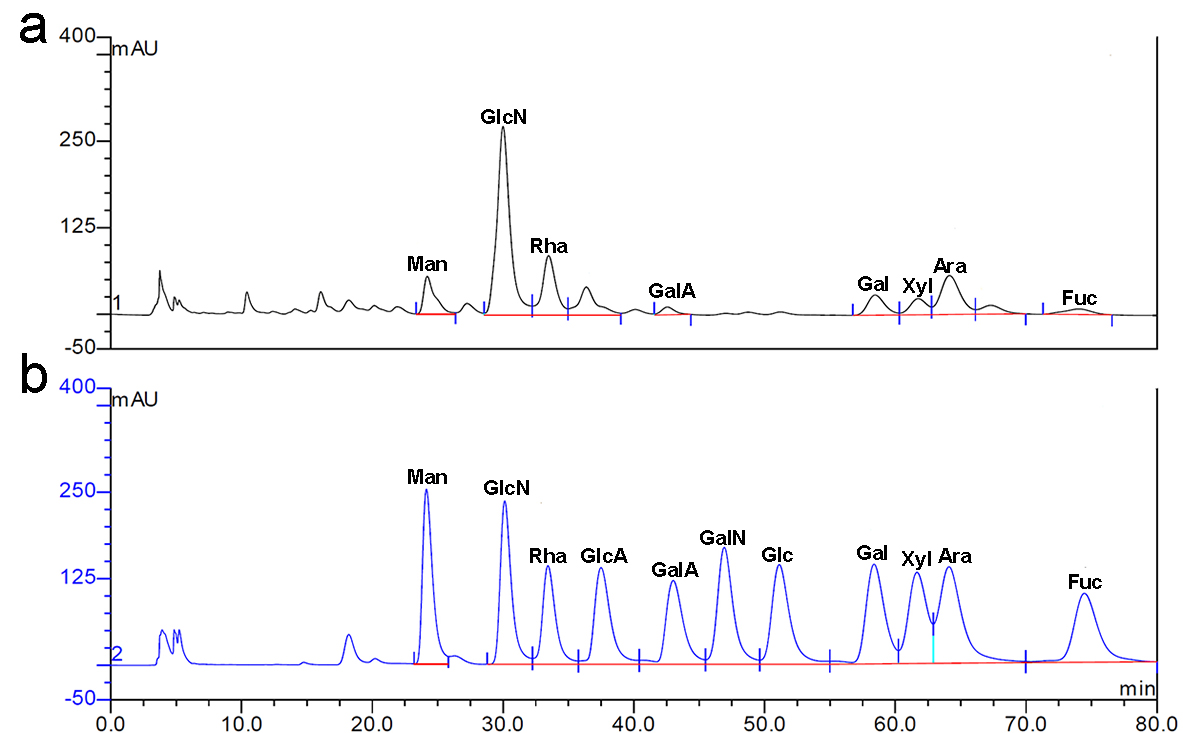


(a) L06vLPS. (b) Standards: mannose (Man), glucosamine (GlcN), rhamnose (Rha), glucose acid (GlcA), galactose acid (GalA), galactosamine (GalN), glucose (Glc), galactose (Gal), xylose (Xyl), arabinose (Ara) and fucose (Fuc).
