# Supplementary material for: A new model of self-resolving leptospirosis in mice infected with a strain of Leptospira interrogans serovar Autumnalis harboring LPS signaling only through TLR4
Source: Emerg Microbes Infect. 2017 May 24;6(5):e36–. doi: 10.1038/emi.2017.16 (PMC5520481; doi:10.1038/emi.2017.16)
Supplement: Supplementary Figure S4 [file emi201716x4.docx]

**Supplementary Figure S4 L06vLPS-induced IL-6 production in PEMs of WT, TLR4^-/-^, and TLR2^-/-^ mice.**


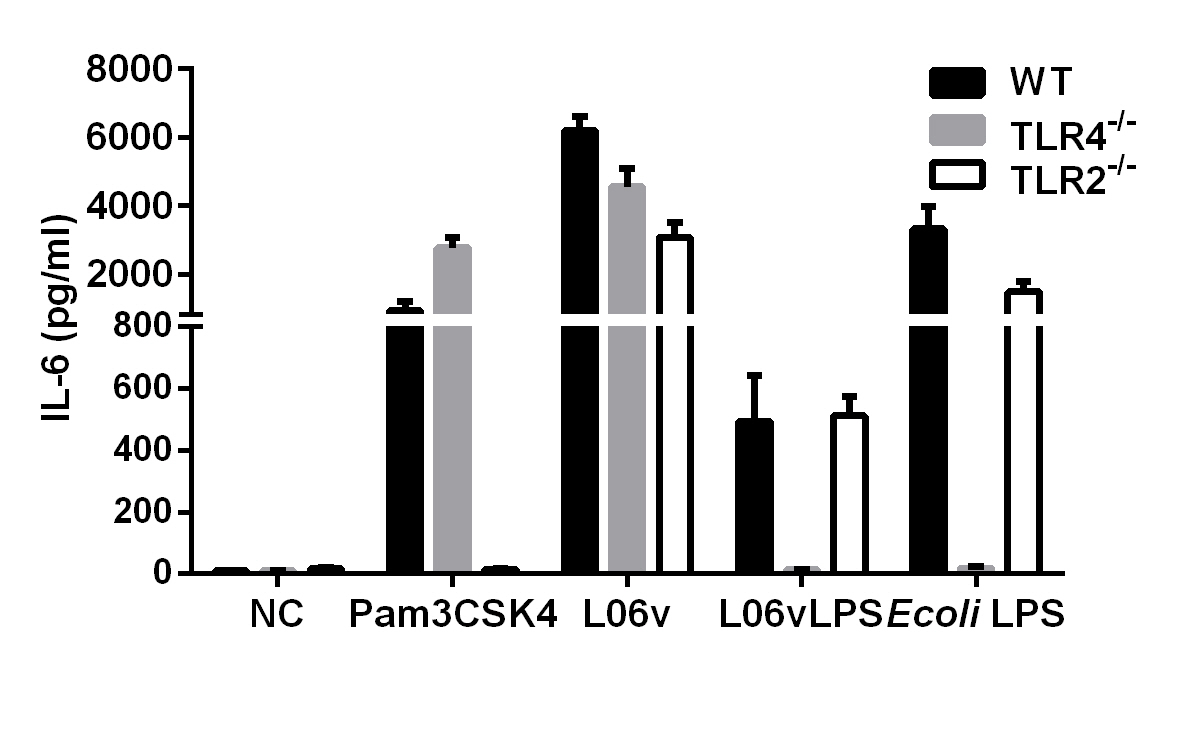


PEMs of WT, TLR4^-/-^, and TLR2^-/-^ mice were stimulated with PBS (NC), *L. interrogans* strain 56606v (L06v, MOI = 50), L06vLPS (10,000 ng/ml), *E. coli* LPS (10 ng/ml) or Pam3CSK4 (300 ng/ml) for 18 h. IL-6 concentrations from cell culture supernatants were measured by ELISA. Data are expressed as the mean ± SD of triplicate samples from one experiment and are representative of at least three independent experiments.
