# Supplementary material for: A new model of self-resolving leptospirosis in mice infected with a strain of Leptospira interrogans serovar Autumnalis harboring LPS signaling only through TLR4
Source: Emerg Microbes Infect. 2017 May 24;6(5):e36–. doi: 10.1038/emi.2017.16 (PMC5520481; doi:10.1038/emi.2017.16)
Supplement: Supplementary Figure S5 [file emi201716x5.docx]

**Supplementary Figure S5 Structure of *rfb* locus of *L. interrogans* strain 56606v.**


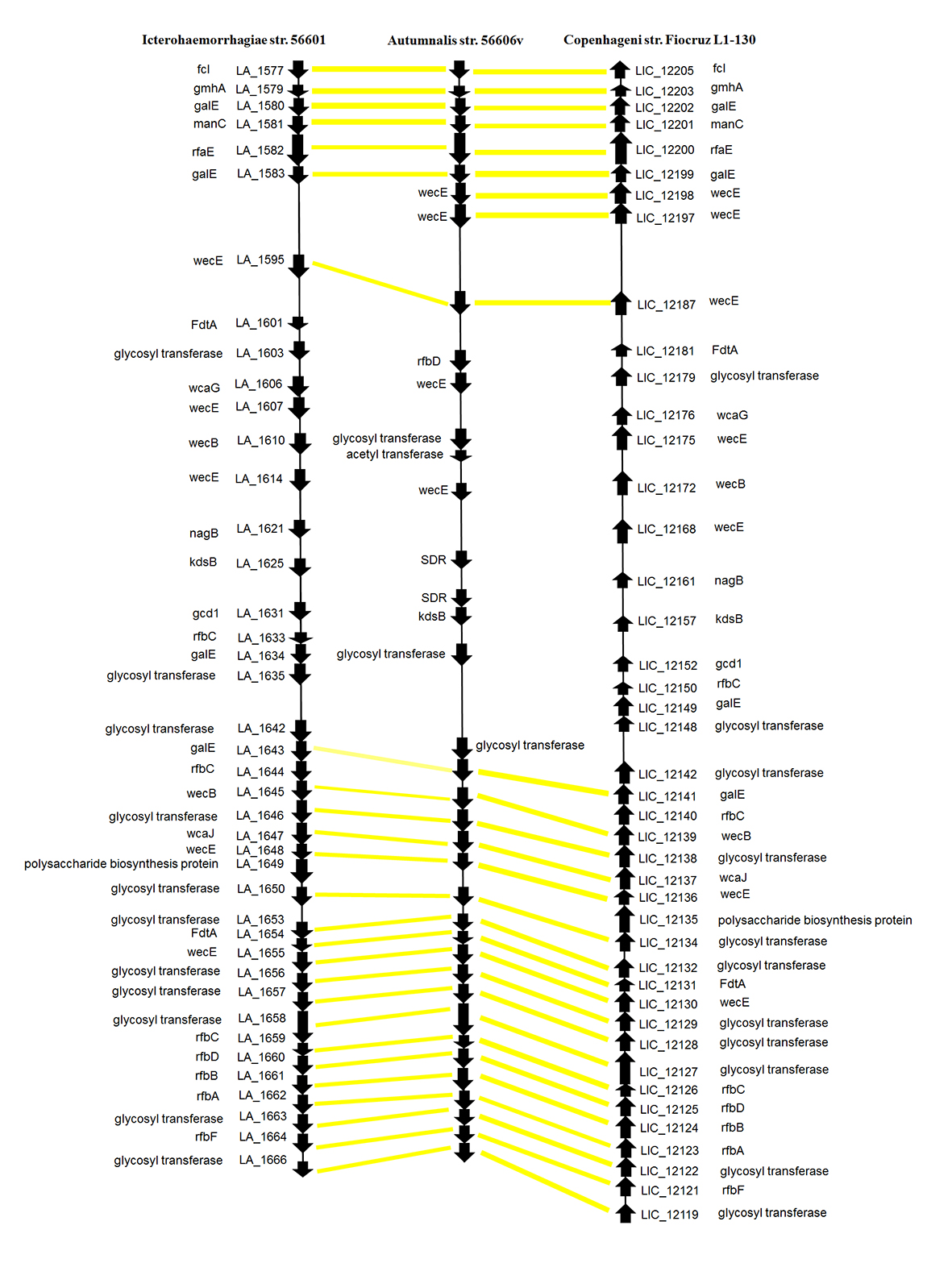


Genes involved in the biosynthesis of the O-antigen within the *rfb* locus of *L. interrogans* serovar Autumnalis are compared to *L. interrogans* serovar Copenhageni strain [Fiocruz L1-130](http://www.ncbi.nlm.nih.gov/genome/179?genome_assembly_id=164786) and serovar Lai strain 56601. Genes involved in the biosynthesis of the O-antigen are shown as arrows. The conserved genes are connected with yellow lines (> 95% identity).
