# Supplementary material for: A new model of self-resolving leptospirosis in mice infected with a strain of Leptospira interrogans serovar Autumnalis harboring LPS signaling only through TLR4
Source: Emerg Microbes Infect. 2017 May 24;6(5):e36–. doi: 10.1038/emi.2017.16 (PMC5520481; doi:10.1038/emi.2017.16)
Supplement: Supplementary Table S1 [file emi201716x6.docx]

**Supplementary Figure Table S1 Scores of liver and lung histopathological lesions after *Leptospira* infection**

|  |  | Liver |  |  | Lung | |
| --- | --- | --- | --- | --- | --- | --- |
| Index | architecture loss | hepatocyte focal necrosis | inflammatory infiltrates |  | intra-alveolar hemorrhage | inflammatory infiltrates |
| WT (NC) | 0, 0, 0 | 0, 0, 0 | 0, 0, 0 |  | 0, 0, 0 | 0, 0, 0 |
| WT | 1.4, 1.5, 1.4 | 0.8, 0.6, 0.9 | 0.7, 0.6, 0.5 |  | 1.9, 2, 1.8 | 1.2, 1.1, 1.1 |
| TLR4^-/-^ | 2.4, 2.5, 2.4 | 1.3, 1.3, 1.4 | 1.1, 0.9, 1 |  | 1.1, 1.2, 1.1 | 1.3, 1.3, 1.2 |

For each mouse, the score of each index for the characterization of tissue injury was calculated by the mean score of ten fields randomly selected from each section. The injury scores in each category are shown in this table.
